# Anti-Proliferative and Pro-Apoptotic vLMW Fucoidan Formulas Decrease PD-L1 Surface Expression in EBV Latency III and DLBCL Tumoral B-Cells by Decreasing Actin Network

**DOI:** 10.3390/md21020132

**Published:** 2023-02-18

**Authors:** Jennifer Saliba, Chanez Manseur, Hugo Groult, Hussein Akil, Mona Tannoury, Danielle Troutaud, Thierry Maugard, Jean Feuillard, Ingrid Arnaudin, Chantal Jayat-Vignoles

**Affiliations:** 1 UMR CNRS 7276, INSERM 1262, CRIBL Laboratory, Faculty of Medecine, Limoges University, 87000 Limoges, France; 2 UMR CNRS 7266, LIENSs Laboratory, La Rochelle University, 17000 La Rochelle, France; 3 Faculty of Sciences II, Lebanese University, Beirut RGHC+4PR, Lebanon; 4 Hematology Laboratory, CHU Dupuytren, 2 Avenue Martin Luther King, CEDEX 87042 Limoges, France

**Keywords:** fucoidan, very-low-molecular-weight fucoidan, PD-L1, EBV latency III B-cells, DLBCLs, actin network

## Abstract

Epstein–Barr virus (EBV) infects 95% of the world’s population and persists latently in the body. It immortalizes B-cells and is associated with lymphomas. LCLs (lymphoblastoid cell lines, EBV latency III B-cells) inhibit anti-tumoral T-cell response following PD-L1 overexpression (programmed death-ligand 1 immune checkpoint). Many cancer cells, including some DLBCLs (diffuse large B-cell lymphomas), also overexpress PD-L1. Immunotherapies are based on inhibition of PD-L1/PD-1 interactions but present some dose-dependent toxicities. We aim to find new strategies to improve their efficiency by decreasing PD-L1 expression. Fucoidan, a polysaccharide extracted from brown seaweed, exhibits immunomodulatory and anti-tumor activities depending on its polymerization degree, but data are scarce on lymphoma cells or immune checkpoints. LCLs and DLBCLs cells were treated with native fucoidan (*Fucus vesiculosus*) or original very-low-molecular-weight fucoidan formulas (vLMW-F). We observed cell proliferation decrease and apoptosis induction increase with vLMW-F and no toxicity on normal B- and T-cells. We highlighted a decrease in transcriptional and PD-L1 surface expression, even more efficient for vLMW than native fucoidan. This can be explained by actin network alteration, suggesting lower fusion of secretory vesicles carrying PD-L1 with the plasma membrane. We propose vLMW-F as potential adjuvants to immunotherapy due to their anti-proliferative and proapoptotic effects and ability to decrease PD-L1 membrane expression.

## 1. Introduction

EBV is an oncogenic virus that infects about 95% of the worldwide adult population. After primo-infection, it remains hidden in nuclei of memory B-cells, resulting in life-long persistent infection. During infection, including transient reactivation, some infected B-cells enter the lytic cycle or EBV latency III program (also called proliferation program), with transcription of the full range of latent genes [1]. In an immunocompetent host, the balance established between the immune system and the virus avoids development of cancers. However, a rupture in equilibrium can occur, causing EBV lymphoproliferative disorders, such as Hodgkin lymphomas (HL), Burkitt lymphomas (BL) or DLBCLs [2], which is the most common non-Hodgkin lymphoma. This aggressive tumor affects B-lymphocytes and has two major biologically distinct subtypes: germinal center B-cell (GCB) and activated B-cell (ABC) [3]. ABC-DLBCL is associated with worse outcomes when treated with chemo-immunotherapy, the standard clinical care for this pathology.

Inhibitory immune checkpoints exert inhibitory effects on adaptive and innate immune systems. They are crucial for self-tolerance but also mediate immune evasion of cancer cells, contributing to tumor emergence and development [4]. PD-L1 (also known as B7-H1 or CD274) is expressed on a sizeable fraction of tumor types and is one of the most critical [5]. It can interact with its receptor, PD-1, which is expressed on numerous cells involved in anti-tumor response (such as activated T-cells, dendritic cells or NK cells) and leads to inhibitory signals [6]. We have previously shown that PD-L1 is also overexpressed on EBV latency III B-cells and strongly inhibits anti-tumoral T-cell response in an autologous B/T model; expansion of anergic conventional and unconventional CD4^+^ Tregs (regulatory T-cells) leads to inhibition of CD4^+^ and CD8^+^ effector T-cells proliferation [7]. The PD-L1/PD-1 axis is also critical for numerous B-cell lymphomas, such as HL and non-Hodgkin lymphomas, among them follicular lymphoma and DLBCL (which variably express PD-L1) [8,9]. PD-L1 overexpression in many cancer types and strong immune response inhibition following interaction with PD-1 have led to development of anti-PD-L1 and anti-PD-1 immunotherapies to block the PD-L1/PD-1 axis and restore immune response [10,11]. However, immune checkpoint inhibitors have some limitations since tolerance breakdown is not limited to tumors; related adverse events can affect multiple organ systems (gut, skin, endocrine glands, liver or lung) [12,13,14]. While keeping the advantage of immunotherapy, new therapeutic strategies can be considered, such as use of adjuvants capable of decreasing PD-L1 membrane expression and consequently lowering antibody doses [15].

Fucoidans are non-allergenic, non-irritating, biodegradable and biocompatible sulfated fucose-based polysaccharides constituents of brown seaweeds [16,17]. Extracts from *Fucus vesiculosus* algae are approved by the FDA (Food and Drug Administration) as GRAS (generally recognized as safe). In Europe, preparations that contain fucoidans are registered by the EMA (European Medicines Agency) for use as ingredients in food categories [17,18]. Numerous studies report bioactive properties with health benefits, predominantly dependent on the natural source extraction method, sulfate groups content and molecular weight of polysaccharide. For instance, fucoidans possess antioxidant, anticoagulant, anti-pathogenic and anti-inflammatory activities [19,20,21]. They also exhibit *in vitro* and *in vivo* anticancer properties, almost exclusively studied on solid tumor cells (colon, breast, lung, bladder, hepatoma or melanoma). The mechanisms of action described are generally cell cycle arrest, apoptosis, anti-metastatic effects and stimulation of macrophages, T-cells and NK cells. Nevertheless, scarce studies indicate decrease in PD-L1 checkpoint [22,23], which can be consistent with the fact that fucoidan impairs pathways responsible for PD-L1 expression: PI3k/AKT, NF-κB or RAS/ERK1/2 [24,25,26,27,28,29]. Fucoidan extracts can also protect against side effects associated with chemotherapeutic drugs and radiation-induced damages [16,30]. As suggested by some authors who evaluated pre-clinical safety of fucoidan extracts [31], they may become an appropriate and natural anticancer therapeutic as an adjunctive antitumor drug. However, more information is needed, particularly in the field of hematological malignancies and immune checkpoints, such as PD-L1, which is the overall objective in this study. We evaluated opportunity to use vLMW-F to decrease PD-L1 membrane expression and associated molecular mechanisms.

## 2. Results

### 2.1. Proliferation Inhibition and Apoptosis Induction of Tumoral B-Cells

**Figure 1:**
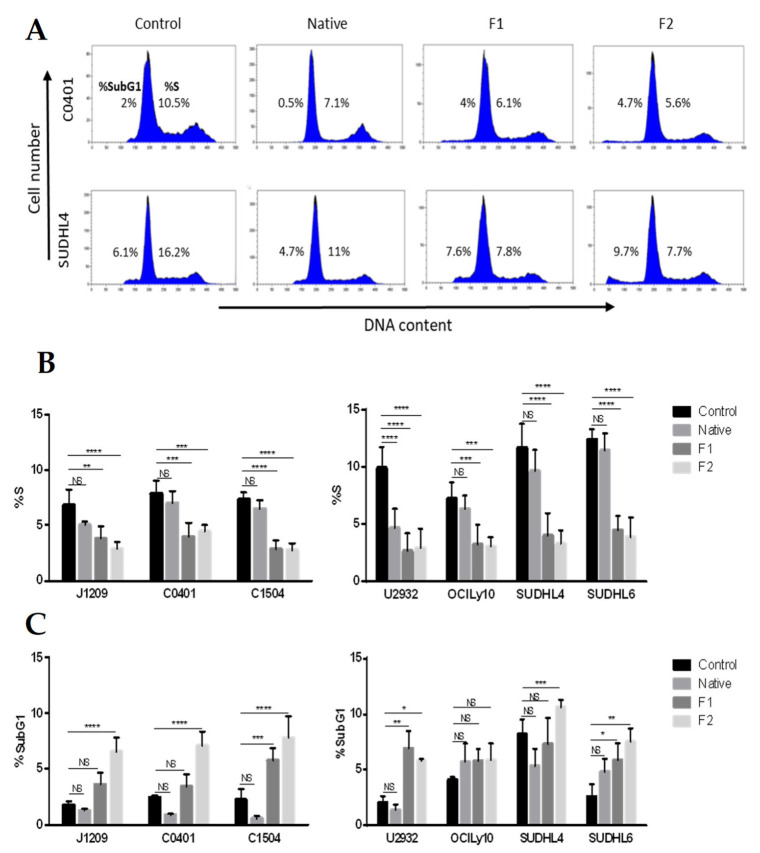
vLMW-F decrease the percentage of LCLs and DLBCLs cells in S-phase. LCLs (J1209, C0401, C1504) and DLBCLs (U2932, OCILy10, SUDHL4, SUDHL6) cells were treated or not (control) with 100µg/mL of native fucoidan or vLMW-F (F1 and F2) for 48 h. Flow cytometry analysis was performed to determine cell-cycle distribution in all tested conditions. Results were obtained from three independent experiments. (**A**) Examples of cell cycle profiles for C0401 (LCL) and SUDHL4 (DLBCL). (**B**) Percentage of LCLs or DLBCLs cells in S-phase: fewer cells are in S-phase after 48 h treatment of 100 µg/mL vLMW-F in contrast with the native form and compared to the control. (**C**) Percentage of LCLs or DLBCLs cells in SubG1 phase: the increase in cell percentage in SubG1 phase occasionally observed suggests apoptosis induction. NS: not significant; * *p*< 0.05; ** *p* < 0.01; *** *p* < 0.001; **** *p* < 0.0001.

Anti-cancer properties of fucoidan (mainly with extracted fucoidan of high molecular weight) were almost exclusively studied on solid tumors and emphasized anti-proliferative and pro-apoptotic events [32]. In order to confirm this role on lymphoma B-cells, we performed cell cycle analysis by flow cytometry on EBV latency III B-cells (three LCLs) and DLBCL cells (two ABCs: U2932 and OCILy10 and two GCBs: SUDHL4 and SUDHL6 cell lines) treated with native or vLMW-F F1 and F2. Estimation of cell population percentage in the different phases of the cell cycle highlighted significant decrease in S phase after treatment with vLMW-F in contrast to the native form (Figure 1A, B). This was consistent with inhibition of cell proliferation. A significant increase in subG1 peak on DNA content histograms (subsequent to fragmentation of nuclear DNA in late apoptosis) was also sometimes observed, depending on cell types, for treatment by vLMW-F, especially F2 (Figure 1C).

**Figure 2:**
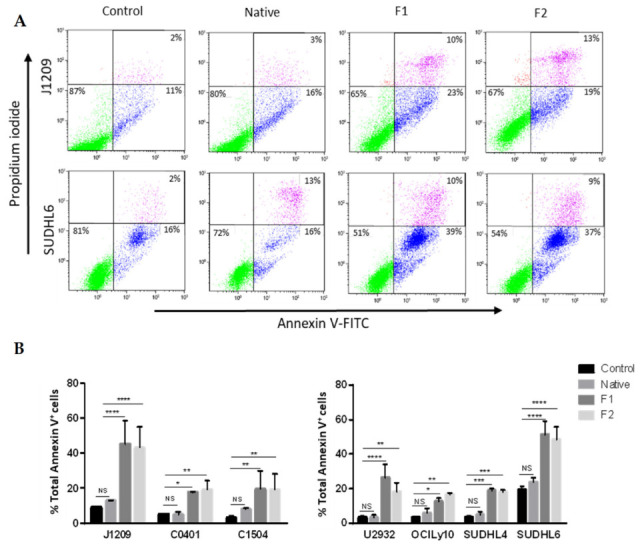
vLMW-F induce apoptosis in LCLs and DLBCLs. LCLs (J1209, C0401, C1504) and DLBCLs (U2932, OCILy10, SUDHL4, SUDHL6) cells were treated or not (control) with 100µg/mL of native fucoidan or vLMW-F (F1 and F2) for 48 h, followed by apoptosis analysis (Annexin V/PI staining) by flow cytometry. Results were obtained from three independent experiments. (**A**) Examples of cell apoptosis for J1209 (LCL) and SUDHL6 (DLBCL) are shown (intact cells: green events–early apoptotic cells: blue events–late apoptotic cells: purple events). (**B**) Percentage of LCLs or DLBCLs total Annexin V+ cells. vLMW-F fractions induce similar apoptosis. NS: not significant; * *p*< 0.05; ** *p* < 0.01; *** *p* < 0.001; **** *p* < 0.0001.

To refine the apoptotic response, we evaluated percentage of apoptotic cells, from early to late stages, for the same cell lines and in the same treatment conditions. Annexin-V binding to externalized phosphatidylserine revealed apoptosis induction only with the two vLMW-F (Figure 2). The importance of the process depended on the cell lines (up to 50% for J1209 and SUDHL6) and was similar for the two fractions. Our results emphasized that, unlike the native form, vLMW-F possess anti-proliferative and pro-apoptotic properties for EBV latency III and DLBCL tumoral cells at the tested dose. This suggests a better cytotoxic effect for vLMW-F. We verified that toxicity (viability and apoptosis) was due to the formulas and not L-Fucose since it is the smallest subunit of fucoidan skeleton (Figure S2).

### 2.2. Absence of Toxicity for Normal B- and T-Cells

**Figure 3:**
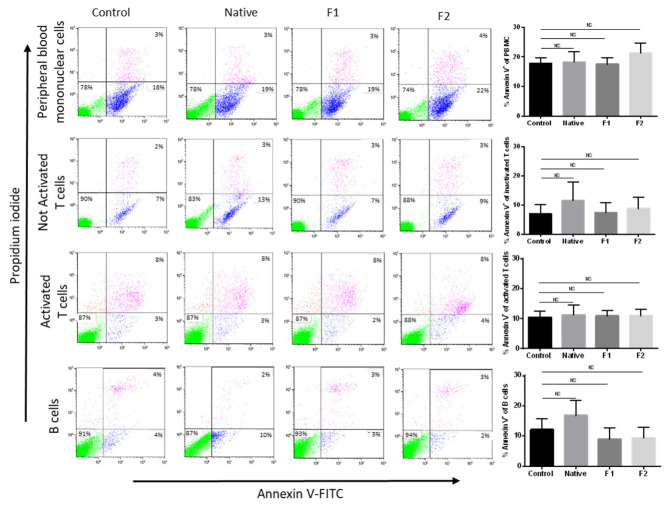
Native fucoidan or vLMW-F are not toxic for normal lymphocyte cells. Normal cells (mononuclear cells, B-cells, T-cells activated or not) were treated or not (control) with 100µg/mL of native fucoidan or vLMW-F (F1 and F2) for 48 h. Apoptosis analysis (Annexin V/PI staining) was realized by flow cytometry. Results were obtained from three independent experiments. An example of each population is shown (intact cells: green events–early apoptotic cells: blue events–late apoptotic cells: purple events) as well as the percentage of total Annexin V^+^ cells for each condition. No apoptosis induction was observed. NS: not significant.

Potential use of native fucoidan or vLMW-F for biomedical application and specificity of the treatment require that they do not display toxicity for normal cells. We chose to focus on peripheral blood mononuclear cells and particularly on normal B-cells (for comparison with tumoral B-cells), T-cells and activated T-cells (frequently implicated in anti-tumor responses via the PD-L1/PD-1 axis). We performed an Annexin V-based flow cytometry test that enables assessing simultaneously apoptosis and viability. No cytotoxic effect was observed for either the native form or the two vLMW-F (Figure 3).

### 2.3. Decrease in PD-L1 Transcriptional Expression

**Figure 4:**
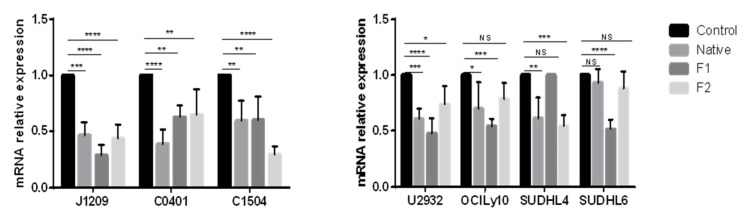
Fucoidan downregulates transcriptional expression of *PD-L1* in LCLs and DLBCLs cells. LCLs (J1209, C0401, C1504) and DLBCLs (U2932, OCILy10, SUDHL4, SUDHL6) were treated with 100µg/mL of native fucoidan or vLMW-F (F1 and F2) for 48 h followed by RNA extraction and RT-qPCR. Results were obtained from three independent experiments. mRNA relative expression of *PD-L1* was decreased either for the native form or the fractions. NS: not significant; * *p*< 0.05; ** *p* < 0.01; *** *p* < 0.001; **** *p* < 0.0001.

*PD-L1* transcriptional expression involves signaling pathways that can be inhibited by fucoidan, such as PI3k/AKT, NF-κB or RAS/ERK1/2 [33,34,35,36]. Therefore, we studied the effect of native and vLMW-F (F1 and F2) on mRNA expression of *PD-L1* for the three LCLs and four DLBCLs cell lines. We showed that it was strongly decreased in LCLs regardless of treatment and to a lesser extent in DLBCLs (Figure 4). Our results emphasized that transcriptional expression of *PD-L1* can be strongly impaired by native fucoidan or vLMW-F.

### 2.4. Decrease of membrane, but Not of total, PD-L1 protein expression 

**Figure 5:**
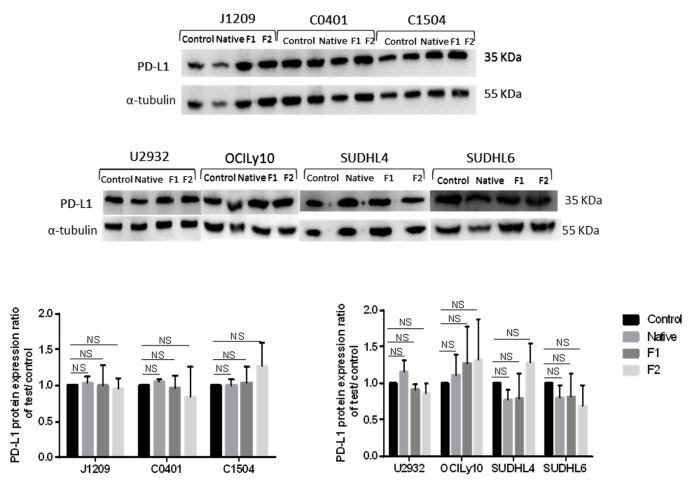
Native fucoidan or vLMW-F do not modify PD-L1 total expression. PD-L1 total expression analysis for LCLs (J1209, C0401, C1504) and DLBCLs (U2932, OCILy10, SUDHL4, SUDHL6) by Western blot after 48 h of 100 µg/mL native fucoidan or vLMW-F (F1 and F2) treatment. No significant change was observed for PD-L1 total expression. Results were obtained from three independent experiments. NS: not significant.

Since mRNA expression can differ from protein expression, especially due to translational regulation, we studied the total protein expression of PD-L1 for the same cell lines and in the same treatment conditions. All the cell lines strongly expressed PD-L1 and, surprisingly, despite inhibition of transcriptional regulation, total PD-L1 expression remained unchanged or occasionally slightly modified whatever the cell line and treatment, as observed by Western blot (Figure 5) or flow cytometry (Figure S3). This could be explained by the fact that PD-L1 is stored in secretory lysosomes before their migration *via* the actin network and their fusion with the plasma membrane, which could mask transcriptional regulation.

**Figure 6:**
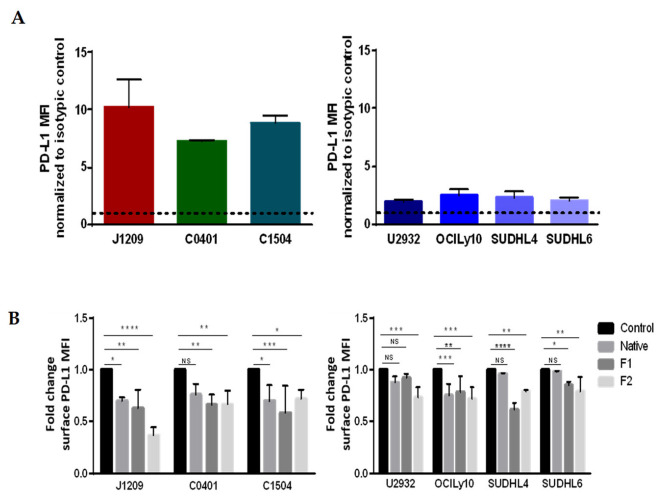
vLMW-F decrease PD-L1 surface expression in LCLs and DLBCLs cells. LCLs (J1209, C0401, C1504) and DLBCLs (U2932, OCILy10, SUDHL4, SUDHL6) cells were treated with 100µg/mL of native fucoidan or vLMW-F (F1 and F2) for 48 h followed by immunofluorescent staining for PD-L1 analyzed by flow cytometry. Results were obtained from three independent experiments. (**A**) PD-L1 is overexpressed by LCLs compared to DLBCLs. (**B**) Fold change (ratio of MFI test/MFI control, both normalized to isotypic control. MFI: mean fluorescence intensity) of PD-L1 surface expression for LCLs and DLBCLs. vLMW-F generally decreased more efficiently than the native form, PD-L1 surface expression for LCLs and DLBCLs. NS: not significant; * *p*< 0.05; ** *p* < 0.01; *** *p* < 0.001; **** *p* < 0.0001.

Since the active fraction of PD-L1 responsible for interaction with PD-1 is that at the cell surface, we have also studied its specific expression. Cytometry analysis was carried out on viable cells populations in order not to consider possible degradation of the protein on dead cells. As expected, cytometry analysis showed overexpression of surface PD-L1, which was much higher for LCLs [37] than for DLBCLs cells [8] (Figure 6A). However, unlike for total expression, fucoidan treatments significantly decreased PD-L1 membrane expression for the three LCLs and four DLBCLs, with generally better results for vLMW-F (Figure 6B). Otherwise, PD-L1 decrease was more important for LCLs than DLBCLs that have, however, lower baseline expression. Our results emphasized a specific decrease in PD-L1 membrane expression in the presence of fucoidan species, especially effective on LCLs when treated with vLMW-F. Since total expression remained unchanged, this could be due to regulation of membrane traffic. We verified that a decrease in membrane PD-L1 expression was due to the fractions of vLMW-F and not L-Fucose since it is the smallest subunit of fucoidan skeleton (Figure S4).

### 2.5. Disruption of Actin Network and Decrease in Secretory Activity

**Figure 7:**
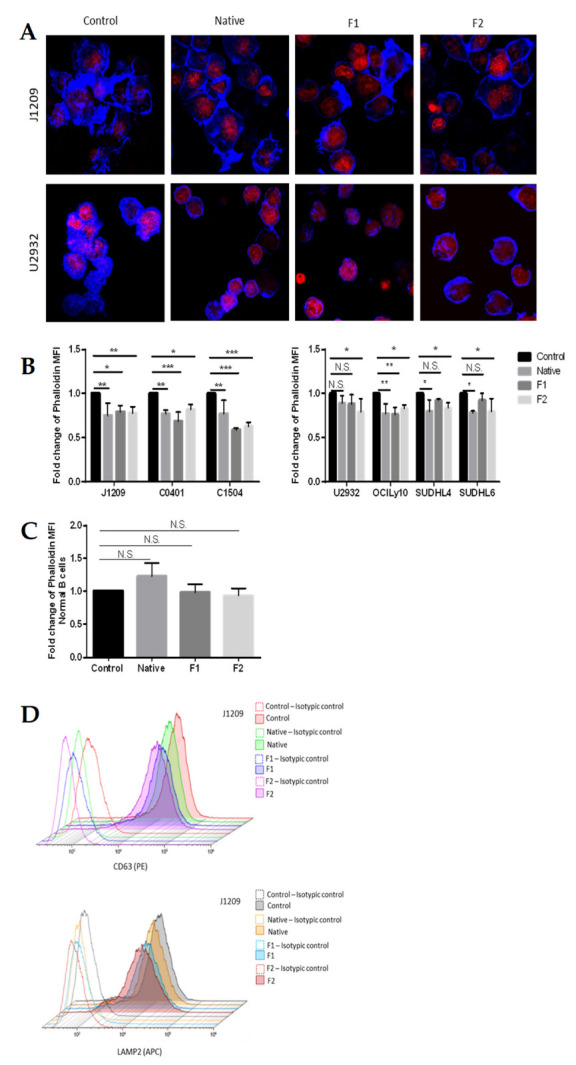
vLMW-F decrease actin polymerization and vesicle markers expression at plasma membrane. (**A**) LCLs (J1209, C0401, C1504) and DLBCLs (U2932, OCILy10, SUDHL4, SUDHL6) cells were treated or not (control) with 100µg/mL of native fucoidan or vLMW-F (F1 and F2) for 48 h, followed by phalloidin staining (F-actin, blue) and TOPRO-3 (nuclei, red) before confocal microscopy observations. (**B**, **C**) Quantification of F-actin in the same conditions analyzed by flow cytometry for (**B**) LCLs and DLBCLs or (**C**) normal B-cells. vLMW-F significantly decreased content of actin polymerization strongly in LCLs and to a lesser extent in DLBCLs but not in normal B-cells. Results were obtained for three independent experiments. (**D**) Flow cytometry fluorescence histograms of surface CD63 and LAMP2 (secretory vesicle markers) for J1209 (obtained for three independent experiments). The decrease is more efficient with vLMW-F than with the native form. NS: not significant; * *p* < 0.05; ** *p* < 0.01; *** *p* < 0.001.

We have already shown for LCLs that PD-L1 membrane expression involved migration of LAMP2^+^/PD-L1^+^ secretory lysosome via the densified actin network and vesicular membrane traffic [37]. Therefore, we studied the impact of native and vLMW-F fractions F1 and F2 on actin network. F-actin was stained by fluorescent phalloidin. We emphasized a marked decrease in actin polymerization, especially for LCLs and to a lesser extent for DLBCLs cells, as visualized by confocal microscopy (Figure 7A) and quantified by flow cytometry (Figure 7B). Furthermore, we showed, by quantifying the F-actin, that, for normal B-cells, neither native fucoidan nor vLMW-F modify actin network (Figure 7C). To assess vesicular membrane traffic, we focused on LCLs, for which PD-L1 membrane expression and actin network were more affected. We studied native fucoidan and vLMW-F effects on two specific reporter molecules, which are constitutive of intracellular vesicles and expressed at the plasma membrane following their fusion: CD63 and LAMP2. Moreover, LAMP2 is otherwise a lysosomal-associated protein expressed by secretory lysosomes (storage location of PD-L1). Analysis by flow cytometry showed a decrease in the two secretory vesicle markers at the plasma membrane for the native fucoidan and even more for vLMW-F F1 and F2 (Figure 7D). Our results suggest that fucoidan impedes secretory activity through the actin network and vesicle fusion with plasma membrane, which leads to decrease in PD-L1 membrane expression.

## 3. Discussion

Our results show that vLMW-F decrease cell proliferation and induce apoptosis of LCLs and DLBCLs tumoral B-cells without being toxic for normal B- and T-cells. Furthermore, these formulas decreased PD-L1 expression at transcriptional and cell surface levels. These results may be explained by actin network alteration that could be associated with deregulation of cells secretory activity, reducing thereby PD-L1 externalization.

Numerous studies report *in vitro* and *in vivo* anti-cancer effects (such as anti-proliferative, pro-apoptotic, anti-angiogenic and anti-metastatic) of fucoidan extracts or their low-molecular-weight derivatives against multiple types of solid tumors, such as colon, breast, hepatocarcinoma, lung or bladder [16,32,38]. On the contrary, scarce data are reported regarding their effects on liquid tumors, especially concerning LMW derivatives of fucoidan. Few studies have shown that native fucoidan extracted from *Fucus vesiculosus* can inhibit proliferation of myeloid and monocytic leukemia cell lines by inducing their apoptosis [24,30,39] or arrest ABC- or GCB-DLBCL cell cycle [40]. Therefore, we first confirmed these effects previously reported on lymphocytic B lineage and demonstrated for the first time that these anti-proliferative and pro-apoptotic activities are also extended to LCLs. We also highlighted that extremely depolymerized formulas (< 600 Da) maintain specific anti-tumor activity with more efficiency than native form while being non-toxic for normal B- and T-cells. This is consistent with the fact that depolymerized forms are generally more potent [21,23] and in agreement with non-toxicity of fucoidans [16,32,41]. Use of depolymerized form enables being more specific with fewer unwanted effects.

We wanted to confirm the role of fucoidans since they, or derivatives, cover a wide range of immunomodulatory effects and can participate in immune response against several cancers and infectious diseases [42,43], sulfate and acetyl groups mainly contributing to the activity [44]. For instance, they have been shown to enhance dendritic cell maturation, cytotoxic T-cell activation, antibody production or memory T-cells production [45,46]. They can also increase production of TNFα by neutrophils *in vitro* and *in vivo*, delaying their apoptosis [45,46], or induce NO synthesis [47] and activate *in vitro* and *in vivo* NK cells [45,48]. However, scarce data exist either for solid or liquid tumors in regard to their effects on the inhibitory immune checkpoints responsible for tumor cells escape from immune surveillance, especially regarding the PD1/PD-L1 axis. Yet, other data have shown that fucoidan impairs pathways responsible for PD-L1 expression, such as PI3k/AKT, NF-κB or RAS/ERK1/2 [33,34,35,36], making them very promising candidates for immune checkpoint modulation. To date, only two articles have reported a native fucoidan that can decrease PD-L1 expression in tumor tissues of experimental-induced mammary cancer [22] and a LMW one that can decrease transcriptional expression of *PD-L1* and *PD-L2* and PD-L1 protein level in fibrosarcoma cells [23]. Thus, it is particularly interesting to better understand effects on PD-L1 since it is particularly critical in immune escape and now of great interest in clinical care/immunotherapy approaches. The decrease in transcriptional expression that we found is in agreement with other studies that have demonstrated that signaling pathways involved in PD-L1 expression (PI3k/AKT, NF-κB or RAS/ERK1/2) can be inhibited by fucoidan [33,34,35,36]. Especially, our results confirm such effect for the first time in B-lymphoma cells, whether with the native form or vLMW-F.

In tumor cells, transcriptional expression of *PD-L1* is often not directly related to protein expression due to stabilizing post-translational modifications, such as N-glycosylation and phosphorylation [49,50]. Moreover, it can be accumulated in vesicles, such as exosomes [5] or secretory lysosomes, as demonstrated for LCLs [37], where we observed cytoplasmic accumulation. The relevant fraction of PD-L1 is that expressed at cell surface and able to interact with inhibitor receptor PD-1 expressed on anti-tumor immune cells. As expected, we observed overexpression for LCLs [37] compared with DLBCLs cells [8]. Interestingly, we next showed a PD-L1 decrease on lymphoma B-cells after treatment by native and our two fucoidan fractions, with little more efficiency for vLMW-F than the native form. It is of importance to note that PD-L1 decrease occurs on viable cells since it reduces their inhibition towards anti-tumor immune cells, while some of them die by apoptosis. The same tests realized with L-fucose monomer showed no effect, clearly demonstrating the real added value for these original vLMW-F.

Maintenance of high intracellular level of PD-L1 and decrease on cell surface emphasized that native fucoidan, as well as vLMW-F, can regulate membrane secretory traffic of this immune checkpoint. A prerequisite for vesicle secretion is their migration close to the plasma membrane *via* the actin network [51,52]. Remodeling and molecular modification of actin network occur in tumor cells [53,54] and constitute a specific and original therapeutic target [55,56]. We previously showed that actin network is increased in LCLs by EBV latency III program and responsible for fusion of secretory lysosome with plasma membrane, leading to PD-L1 overexpression at the cell surface [37]. Presently, we show that native fucoidan, as well as vLMW-F, decrease actin polymerization for LCLs and to a lesser extent for DLBCLs cells, which can be associated with reduction in PD-L1 membrane expression. This is also supported by decrease in LAMP2 membrane expression, particularly after treatment with vLMW-F, which has been correlated with vesicles secretory activity of cells. Data in the literature confirm some links between fucoidan and actin pathway, such as decrease in expression of genes implicated in polymerization, organization and stabilization [57] or disruption of F-actin stress fibers [58,59]. In this context and as a first hypothesis, the difference between native and vLMW forms might be explained by variation in the internalized amount of the different species, especially for their molecular weight differences. No effect on normal cells, associated with lack of toxicity, suggests very interesting specific sensitivity for tumoral actin network. Our results also suggest that expression of other inhibitory immune checkpoints could be decreased insofar as they pass through secretory vesicles [4], secretory lysosomes [37,60] or exosomes [61,62].

As native fucoidan is mainly composed of fucose and because the F1 and F2 fractions present very low DP, such formulas may comprise mono-, di- or tri-saccharides of fucose that are imputable of the effects observed on our cellular models. Results obtained with the single L-fucose monosaccharide did not show any effect. This suggests that F1 and F2 present other original fucoidan-derived compounds of vLMW responsible for these bioactivities, perhaps including glucuronic acid, galactose and xylose (the other principal sugar found in fucoidan) [63], or backbone modifications because of the depolymerization method (opening or creation of insaturation because of H_2_O_2_-based hydrolysis) [64].

Altogether, our results suggest that efficiency of vLMW-F is correlated to their low molecular weight (by comparison with the native form), which is consistent with the literature [23,65]. It will be interesting to study if this can be associated with different capacities of internalization. However, no noteworthy differences were observed between the two fractions, F1 and F2, that differ mainly by their sulfation degree. These slight differences in sulfation percentage could explain the different effects observed with the two vLMW-F in some experiments. Negatively charged sulfate groups are responsible for interaction with numerous molecules and involved in various biological process. They contribute to fucoidan activity. However, at a very low fraction size, this parameter does not seem to be a key factor in the bioactivities observed. Considering their very small size, high internalization of vLMW-F could explain that a lower degree of sulfation is sufficient for optimal activity. Establishing a complete picture of the structure (Mn and % S)/bioactivity relationship of such a complex fucoidan molecular structure is tedious as both parameters can differently influence the outcome of the effect according to the bioactivity sought and the experimental model used. However, in perspective, it could be interesting to compare the results obtained in this work with ones of desulfated native fucoidan and/or oversulfated vLMW-F.

In translational medicine, fucoidan extracts are considered of great interest as adjuvants for cancer therapy [66,67]; benefits are also observed with anti-PD-L1 or anti-PD-1 immunotherapies in mice models. Fucoidan extracted from *Fucus vesiculosus* promoted activation of tumor-infiltrating CD8^+^ T-cells and strongly inhibited growth of melanoma cells when co-administrated with anti-PD-1, particularly when applied before immunotherapy [35]. Fucoidan extracted from three species of algae, among them *Fucus vesiculosus*, promoted proliferation and activity of *ex vivo* PBMCs and potentiated anti-PD-1 effects [68]. Intranasal administration of *Ecklonia-cava*-extracted fucoidan enhanced anti-PD-L1 mediated anti-cancer activities against melanoma and carcinoma tumor growth in lungs [69]. Fucoidan of *Luminaria japonica* enhanced anticancer efficacy of anti-PD-L1 antibodies against Lewis lung carcinoma [70].

Algal polysaccharides, such as fucoidan, could provide novel therapeutic alternatives and promising supplements, especially for cancer treatments. Clinical trials are actually in course for hepatocellular carcinoma (NCT04066660) and rectal cancer (NCT04342949). It could be useful combined with immune checkpoint blockade therapies to treat lymphoproliferative malignancies.

Our results suggest that vLMW-F (<600 Da) could be effective potential adjuvants of anti-PD-L1 or anti-PD-1 immunotherapy thanks to their favorable anti-proliferative and pro-apoptotic effects associated with their ability to decrease membrane PD-L1 *via* actin depolymerization. Since fucoidan possesses anti-inflammatory properties, which could interfere with antitumoral functions (reduction in recruitment of antitumoral killing cells and increase in cancer cell apoptosis and chemo-sensitivity), it will be important to further investigate this activity for vLMW-F. Otherwise, the PD-L1/PD-1 axis plays a crucial role in the tumor microenvironment and interactions between antitumor and cancerous cells. Therefore, functional *in vitro* studies on co-culture models with autologous T-cells or NK cells and *in vivo* studies on mice models are the next steps. Treatments with anti-PD-L1 or anti-PD-1 antibodies and fucoidan (native and vLMW-F) remain to be evaluated, independently and in combination.

## 4. Materials and Methods

### 4.1. Fucoidan Samples

Native fucoidan of *Fucus vesiculosus* was obtained from Sigma-Aldrich. Original formulas were depolymerized by our collaborators from LIENSs laboratory (UMR CNRS 7266, La Rochelle University, France) from the native fucoidan using a radical H_2_O_2_-based hydrolysis method previously published with other types of polysaccharides [71]. Briefly, native fucoidan was dissolved in Milli-Q water (25 mg/mL), and then the solution was purged with argon and heated until 60°C. Addition of H_2_O_2_ 30% (Sigma-Aldrich) at a weight/weight ratio of 0.5 and 1.5 resulted in production of two different vLMW-F fractions, named F1 and F2, respectively, after 96 h and 72 h of depolymerization. The chromatographic profile shows that F1 and F2 fractions are practically eluted at the same time and after the native fucoidan, which attests that the H_2_O_2_-based hydrolysis reaction worked well (Figure S1). Number-average molecular weights (Mn), degree of polymerization (DP) and polydispersity index (I) were estimated by SEC-HPLC according procedures already published [71] using calibrant curves made of pullulans standards (Polymer Standards Service GmbH, Mainz, Germany) for native fucoidan and heparin standards (Iduron, UK) for F1 and F2 fractions. Degree of sulfation (DS) was calculated by an Azure-A-based-colorimetric assay (Sigma-Aldrich) according to a state-of-the-art technique. Results of these characterizations are summarized in Table 1.

As shown in Table 1, the F1 and F2 fractions contain a mixture of different fucoidan-derived compounds with similar very-low-mean DPs between 3.4 and 3.5 but are distinct by their mean sulfation degree, 6% against 2.1%, respectively. Further, LC–MS (data not shown) confirmed that these fractions contain predominantly di- and tri-oligofucoidan with, interestingly, several unsaturated species.

### 4.2. Cell Culture Conditions

Lymphoblastoid cell lines (LCLs–J1209, C0401, C1504) were established and characterized by the Genethon (Evry, France). They were cultured in RPMI 1640 medium (Eurobio Scientific) supplemented with 10% decomplemented FBS (PAN^TM^ Biotech). Amino acids, vitamins, sodium pyruvate, penicillin/streptomycin and 2 mM L-glutamine were added at 1× concentrations from 100× stock solutions (all from Gibco, ThermoFisher). Four cell lines of DLBCLs, two ABC subtypes (U2932 and OCILy10) and two GCB subtypes (SUDHL4 and SUDHL6), were cultured in RPMI 1640 medium supplemented with 10% decomplemented FBS, pyruvate (1×), Penicillin/Streptomycin (1×), L-glutamine (1X) and 10 mM of HEPES buffer solution (Gibco ThermoFisher). All cell lines were maintained at 37 °C in a humified 5% CO_2_ atmosphere and were mycoplasma-free (MycoAlert Mycoplasma Detection Kit). Samples from healthy subjects were obtained from the University Hospital Center of Limoges after their informed consent.

### 4.3. Cell Cycle Analysis

LCLs and DLBCLs cells were seeded in plates (5 × 10^5^ cells/well) for 24 h and then treated with 100 µg/mL of native fucoidan or F1/F2 fractions. After 48 h, cells were collected, washed twice in Dulbecco’s Phosphate Buffered Saline (DPBS–Eurobio Scientific) and fixed with ice-cold 70% ethanol overnight. For Propidium Iodide (PI–Sigma Life Sciences) staining, fixed cells were washed twice with cold DPBS and incubated in 30µL of RNase working solution (10 mg/mL) and 1 mL cold DPBS for 20 min at room temperature (RT). Then, samples were stained with PI and analyzed using a BD FACSCalibur flow cytometer and Kaluza Analysis 2.1 Software (Beckman Coulter).

### 4.4. Apoptosis Analysis

We followed the same protocol of seeding and treatments as described above for cell cycle analysis. LCLs and DLBCLs (5 × 10^5^ cells/well) cells were collected and washed with DPBS containing Ca^2+^. Then, they were stained with Annexin V-FITC (Biolegend) and PI (5µg/mL) for 15 min, in the dark, at RT. Stained cells were analyzed using a BD FACSCalibur flow cytometer and Kaluza Analysis 2.1 Software.

### 4.5. Isolation of Healthy PBMC and Cell Subtypes (B- and T-Cells) for Apoptosis Assay

Isolated peripheral blood mononuclear cells (PBMCs) from all healthy donors were obtained after written consent and were issued from the cell biological collection of the Tissue and Cell Bank CRBioLim of the Limoges Hospital University Center, this cell collection being declared to and authorized by the French Health Ministry with session n° “AC-2021-4790” according to French law. PBMCs were isolated from leukocyte buffy coats by lymphocyte medium separation (MSL, Eurobio Scientific) density gradient centrifugation. T-cells were purified from PBMC by CD3/CD4 EasySep^TM^ human T-cell isolation kit (STEMCELL Technologies) according to the manufacturer’s instructions. Activated T lymphocytes were obtained using T-cell activation/expansion kit (Anti-Biotin MACSiBead Particles and biotinylated antibodies against human CD2, CD3 and CD28) according to the manufacturer’s protocol (Miltenyi Biotec). All cell subtypes were seeded in plates (5 × 10^5^ cells/well) and, after 24 h, were treated or not with 100µg/mL of native fucoidan or vLMW-F. After 48 h, Annexin V/PI staining was performed for cell apoptosis analysis as described above for all cells groups. For flow cytometry analysis, B-cells were identified from mononuclear cells by staining with anti-CD19 (APC) conjugated antibody (Biolegend). The different antibodies and conjugated fluorochromes, as well as final dilutions, are listed in Table S1.

### 4.6. RNA Extraction, Reverse Transcriptase and Real-Time Quantitative PCR

Total RNA was extracted using TRIzol reagent (Life Technologies) from 10^6^ LCLs and DLBCLs cells treated or not. Total RNA (1 µg) was reverse transcribed using the high capacity cDNA Reverse Transcription Kit (Applied Biosystems) according to the manufacturer’s instructions, with 20µL of final reaction volume. Quantitative mRNA relative expression of PD-L1 was performed in triplicate, with 50 ng cDNA, using the Taqman Assay Gene Expression system of PD-L1 (Hs01125296_m1) or GAPDH–internal control–(Hs02758991_g1) (both from ThermoFisher Scientific) with SensiFast Probe HiRox Mix (Bioline), on a Quant Studio3 cycler. Each quantitative PCR was performed in triplicate. The expression level of each gene was normalized to the GAPDH expression level. The calculated relative mRNA expression level was equal to 2^-ΔΔCt^ with untreated cells (control) as reference.

### 4.7. Western Blot Analysis

Control or treated groups were dry pelleted after 48 h of treatment and lysed with equal volumes of 1× lysis buffer (1 mM PMSF and 1 X protease Inhibitor Cocktail) on ice for 30 min. Then, they were sonicated and centrifuged at 18000 G for 20 min at 4 °C. Protein concentrations were determined by Bradford protein assay. Equal amounts of proteins (30 µg) were separated by 12% SDS PAGE gel electrophoresis and then transferred to PVDF membranes that were blocked in PBS 5% BSA containing 0.1% Tween 20 at room temperature for 1 h. Afterward, the membranes were incubated with primary antibodies against PD-L1 (1:200) (Santa Cruz: Biotechnology) or α-tubulin (1:5000) (Cell Signaling) overnight at 4 °C. The next day, membranes were washed (PBS-0.1% Tween) and incubated with HRP-secondary antibody (1:5000) at room temperature for 1 h. After washing, the protein bands were detected with a chemiluminescence detection system (ChemiDoc^TM^ Touch Gel Imaging System—Bio-Rad Laboratories), which were quantified and numerated using Fiji software (Rasband, W.S., ImageJ). A ratio was calculated for PD-L1 expression/α-tubulin expression, and then a second ratio was calculated for test/control to compare expression of treated to untreated cells.

### 4.8. PD-L1 Expression Analysis: Immunofluorescent Staining and Flow Cytometry

For surface labeling, the same protocol of seeding and treatment as described above for cell cycle analysis was followed. LCLs and DLBCLs cells were collected and washed with DPBS. Then, they were labeled for 15 min in the dark at RT with anti-PD-L1-PE (Biolegend) (Table S1). Intracellular PD-L1 staining was performed on LCLs and DLBCLs cells treated with native fucoidan or F1/F2 fractions using the IntraPrep Permeabilization Reagent kit (Beckman Coulter) according to the protocol recommended by the supplier. Acquisitions were performed on FACSCalibur. Results were analyzed with Kaluza Analysis 2.1 Software. Fold change was calculated based on the mean fluorescence intensity ratio of PD-L1 on its isotypic control, and then normalized to the control (untreated cells).

### 4.9. F-Actin Cytoskeleton Immunofluorescence

Following the same experimental protocol of treatment as described above, LCLs and DLBCLs cells were collected and washed with DPBS. After washing, they were fixed with 4% paraformaldehyde for 10 min at RT and washed with DPBS. Actin fibers were revealed using 405-Phalloidin-iFluor reagent (Abcam) as per the manufacturer’s instructions.

Finally, after 30 min of phalloidin incubation, nuclei were stained with TOPRO-3 (1:1000—Fisher scientific) for 15 min at RT. Cells were visualized using a ZEISS LSM 900 confocal microscope (40 × oil lens). Images were constructed using the Fiji software. For actin quantification by flow cytometry, the same experimental protocol was followed. Intracellular actin fibers labeling (45 min of phalloidin staining) was performed after permeabilization (IntraPrep Permeabilization Reagent kit–Beckman Coulter) according to the protocol recommended by the supplier. Acquisitions were performed on the Cytoflex cytometer (Beckman Coulter). Results were analyzed with Kaluza Analysis 2.1 Software. Fold change was calculated based on the mean fluorescence intensity ratio of phalloidin of the test normalized to the control.

### 4.10. Statistical Analysis

One-way or two-way analysis of variance (ANOVA) and *t*-test were performed to identify significant differences between the control and experimental groups. All experimental data were acquired from at least three independent experiments. All statistical analyses were performed with GraphPad Prism 6.05 for Windows. A probability (*p*) value of <0.05 was considered statistically significant.

## Figures and Tables

**Table 1:** Characteristics of native fucoidan and vLMW-F (F1 and F2). Number-average molecular weights (Mn), degree of polymerization (DP), polydispersity index (I) and degree of sulfation (DS)**.** *: calculated with pullulans standards. **: calculated with heparin standards.

Sample	H_2_O_2_ (*w*/*w*)	Time (h)	Mn (Da)	DP	I	DS (% SO_3_^−^)
Native fucoidan	0	0	66744 *	240.5 *	1.4 *	41.5 ± 0.7
F1 fraction	0.5	96	604 **	3.5 **	1.3 **	6.0 ± 1.0
F2 fraction	1.5	72	562 **	3.4 **	1.1 **	2.1 ± 0.4

## Data Availability

The datasets generated and analyzed during the current study are available from the corresponding author on reasonable request.

## Supplementary Materials

The following supporting information can be downloaded at: https://www.mdpi.com/article/10.3390/md21020132/s1, Table S1: Antibodies used for flow cytometry; Figure S1: SEC-HPLC analysis with refractive detector of native fucoidan with F1- and F2-produced fractions; Figure S2: L-fucose does not induce apoptosis in LCLs and DLBCLs; Figure S3: Native fucoidan or vLMW-F do not (or slightly) change PD-L1 total expression; Figure S4: L-fucose does not decrease PD-L1 surface expression in LCLs and DLBCLs cells.

## References

[1] Rowe M., Raithatha S., Shannon-Lowe C. (2014). Counteracting Effects of Cellular Notch and Epstein-Barr Virus EBNA2: Implications for Stromal Effects on Virus-Host Interactions. J. Virol..

[2] Shannon-Lowe C., Rickinson A. (2019). The Global Landscape of EBV-Associated Tumors. Front. Oncol..

[3] Susanibar-Adaniya S., Barta S.K. (2021). 2021 Update on Diffuse large B cell lymphoma: A review of current data and potential applications on risk stratification and management. Am. J. Hematol..

[4] He X., Xu C. (2020). Immune checkpoint signaling and cancer immunotherapy. Cell Res..

[5] Cha J.-H., Chan L.-C., Li C.-W., Hsu J.L., Hung M.-C. (2019). Mechanisms Controlling PD-L1 Expression in Cancer. Mol. Cell.

[6] Wu Y., Chen W., Xu Z.P., Gu W. (2019). PD-L1 Distribution and Perspective for Cancer Immunotherapy—Blockade, Knockdown, or Inhibition. Front. Immunol..

[7] Auclair H., Ouk-Martin C., Roland L., Santa P., Mohamad H.A., Faumont N., Feuillard J., Jayat-Vignoles C. (2019). EBV Latency III–Transformed B Cells Are Inducers of Conventional and Unconventional Regulatory T Cells in a PD-L1–Dependent Manner. J. Immunol..

[8] Boussiotis V.A. (2015). Cell-specific PD-L1 expression in DLBCL. Blood.

[9] Yamamoto R., Nishikori M., Kitawaki T., Sakai T., Hishizawa M., Tashima M., Kondo T., Ohmori K., Kurata M., Hayashi T. (2008). PD-1-PD-1 ligand interaction contributes to immunosuppressive microenvironment of Hodgkin lymphoma. Blood.

[10] Gu D., Ao X., Yang Y., Chen Z., Xu X. (2018). Soluble immune checkpoints in cancer: Production, function and biological significance. J. Immunother. Cancer.

[11] Khan M., Arooj S., Wang H. (2021). Soluble B7-CD28 Family Inhibitory Immune Checkpoint Proteins and Anti-Cancer Immunotherapy. Front. Immunol..

[12] Conroy M., Naidoo J. (2022). Immune-related adverse events and the balancing act of immunotherapy. Nat. Commun..

[13] Michot J.M., Bigenwald C., Champiat S., Collins M., Carbonnel F., Postel-Vinay S., Berdelou A., Varga A., Bahleda R., Hollebecque A. (2016). Immune-related adverse events with immune checkpoint blockade: A comprehensive review. Eur. J. Cancer.

[14] Palmieri D.J., Carlino M.S. (2018). Immune Checkpoint Inhibitor Toxicity. Curr. Oncol. Rep..

[15] Wu M., Huang Q., Xie Y., Wu X., Ma H., Zhang Y., Xia Y. (2022). Improvement of the anticancer efficacy of PD-1/PD-L1 blockade via combination therapy and PD-L1 regulation. J. Hematol. Oncol..

[16] Jin J.-O., Chauhan P.S., Arukha A.P., Chavda V., Dubey A., Yadav D. (2021). The Therapeutic Potential of the Anticancer Activity of Fucoidan: Current Advances and Hurdles. Mar. Drugs.

[17] Citkowska A., Szekalska M., Winnicka K. (2019). Possibilities of Fucoidan Utilization in the Development of Pharmaceutical Dosage Forms. Mar. Drugs.

[18] (2014). Committee on Herbal Medicinal Products. Assessment Report on Fucus vesiculosus L., Thallus.

[19] Cumashi A., Ushakova N.A., Preobrazhenskaya M.E., D’Incecco A., Piccoli A., Totani L., Tinari N., Morozevich G.E., Berman A.E., Bilan M.I. (2007). A comparative study of the anti-inflammatory, anticoagulant, antiangiogenic, and antiadhesive activities of nine different fucoidans from brown seaweeds. Glycobiology.

[20] Fitton J.H., Stringer D.N., Karpiniec S.S. (2015). Therapies from Fucoidan: An Update. Mar. Drugs.

[21] Wang Y., Xing M., Cao Q., Ji A., Liang H., Song S. (2019). Biological Activities of Fucoidan and the Factors Mediating Its Therapeutic Effects: A Review of Recent Studies. Mar. Drugs.

[22] Xue M., Liang H., Tang Q., Xue C., He X., Zhang L., Zhang Z., Liang Z., Bian K., Zhang L. (2017). The Protective and Immunomodulatory Effects of Fucoidan Against 7,12-Dimethyl benz[a]anthracene-Induced Experimental Mammary Carcinogenesis Through the PD1/PDL1 Signaling Pathway in Rats. Nutr. Cancer.

[23] Teruya K., Kusumoto Y., Eto H., Nakamichi N., Shirahata S. (2019). Selective Suppression of Cell Growth and Programmed Cell Death-Ligand 1 Expression in HT1080 Fibrosarcoma Cells by Low Molecular Weight Fucoidan Extract. Mar. Drugs.

[24] Jin J.-O., Song M.-G., Kim Y.-N., Park J.-I., Kwak J.-Y. (2010). The mechanism of fucoidan-induced apoptosis in leukemic cells: Involvement of ERK1/2, JNK, glutathione, and nitric oxide. Mol. Carcinog..

[25] Lee H., Kim J.-S., Kim E. (2012). Fucoidan from Seaweed Fucus vesiculosus Inhibits Migration and Invasion of Human Lung Cancer Cell via PI3K-Akt-mTOR Pathways. PLoS ONE.

[26] Boo H.-J., Hong J.-Y., Kim S.-C., Kang J.-I., Kim M.-K., Kim E.-J., Hyun J.-W., Koh Y.-S., Yoo E.-S., Kwon J.-M. (2013). The Anticancer Effect of Fucoidan in PC-3 Prostate Cancer Cells. Mar. Drugs.

[27] Liu S., Yang J., Peng X., Li J., Zhu C. (2020). The Natural Product Fucoidan Inhibits Proliferation and Induces Apoptosis of Human Ovarian Cancer Cells: Focus on the PI3K/Akt Signaling Pathway. Cancer Manag. Res..

[28] Han Y., Lee J.H., Lee S.H. (2015). Antitumor Effects of Fucoidan on Human Colon Cancer Cells via Activation of Akt Signaling. Biomol. Ther..

[29] Chen J., Jiang C.C., Jin L., Zhang X.D. (2016). Regulation of PD-L1: A novel role of pro-survival signalling in cancer. Ann. Oncol..

[30] Atashrazm F., Lowenthal R.M., Woods G.M., Holloway A.F., Dickinson J.L. (2015). Fucoidan and Cancer: A Multifunctional Molecule with Anti-Tumor Potential. Mar. Drugs.

[31] Mathew L., Burney M., Gaikwad A., Nyshadham P., Nugent E.K., Gonzalez A., Smith J.A. (2017). Preclinical Evaluation of Safety of Fucoidan Extracts from *Undaria pinnatifida* and Fucus vesiculosus for Use in Cancer Treatment. Integr. Cancer Ther..

[32] Lin Y., Qi X., Liu H., Xue K., Xu S., Tian Z. (2020). The anti-cancer effects of fucoidan: A review of both in vivo and in vitro investigations. Cancer Cell Int..

[33] Reyes M.E., Riquelme I., Salvo T., Zanella L., Letelier P., Brebi P. (2020). Brown Seaweed Fucoidan in Cancer: Implications in Metastasis and Drug Resistance. Mar. Drugs.

[34] Vincent-Fabert C., Roland L., Zimber-Strobl U., Feuillard J., Faumont N. (2019). Pre-clinical blocking of PD-L1 molecule, which expression is down regulated by NF-κB, JAK1/JAK2 and BTK inhibitors, induces regression of activated B-cell lymphoma. Cell Commun. Signal..

[35] Yang J., Yang X., Pan W., Wang M., Lu Y., Zhang J., Fang Z., Zhang X., Ji Y., Bei J.-X. (2021). Fucoidan-Supplemented Diet Potentiates Immune Checkpoint Blockage by Enhancing Antitumor Immunity. Front. Cell Dev. Biol..

[36] Ye J., Chen D., Ye Z., Huang Y., Zhang N., Lui E.M.K., Xue C., Xiao M. (2020). Fucoidan Isolated from Saccharina japonica Inhibits LPS-Induced Inflammation in Macrophages via Blocking NF-κB, MAPK and JAK-STAT Pathways. Mar. Drugs.

[37] Durand-Panteix S., Farhat M., Youlyouz-Marfak I., Rouaud P., Ouk-Martin C., David A., Faumont N., Feuillard J., Jayat-Vignoles C. (2012). B7-H1, which represses EBV-immortalized B cell killing by autologous T and NK cells, is oppositely regulated by c-Myc and EBV latency III program at both mRNA and secretory lysosome levels. J. Immunol..

[38] Tsai H.-L., Tai C.-J., Huang C.-W., Chang F.-R., Wang J.-Y. (2017). Efficacy of Low-Molecular-Weight Fucoidan as a Supplemental Therapy in Metastatic Colorectal Cancer Patients: A Double-Blind Randomized Controlled Trial. Mar. Drugs.

[39] Park H.S., Hwang H.J., Kim G.-Y., Cha H.-J., Kim W.-J., Kim N.D., Yoo Y.H., Choi Y.H. (2013). Induction of apoptosis by fucoidan in human leukemia U937 cells through activation of p38 MAPK and modulation of Bcl-2 family. Mar. Drugs.

[40] Yang G., Zhang Q., Kong Y., Xie B., Gao M., Tao Y., Xu H., Zhan F., Dai B., Shi J. (2015). Antitumor activity of fucoidan against diffuse large B cell lymphoma in vitro and in vivo. Acta Biochim. Biophys. Sin..

[41] Hwang P.-A., Yan M.-D., Lin H.-T.V., Li K.-L., Lin Y.-C. (2016). Toxicological Evaluation of Low Molecular Weight Fucoidan In Vitro and In Vivo. Mar. Drugs.

[42] Apostolova E., Lukova P., Baldzhieva A., Katsarov P., Nikolova M., Iliev I., Peychev L., Trica B., Oancea F., Delattre C. (2020). Immunomodulatory and Anti-Inflammatory Effects of Fucoidan: A Review. Polymers.

[43] Hwang J., Yadav D., Lee P.C., Jin J.-O. (2022). Immunomodulatory effects of polysaccharides from marine algae for treating cancer, infectious disease, and inflammation. Phytother. Res..

[44] Ferreira S.S., Passos C.P., Madureira P., Vilanova M., Coimbra M.A. (2015). Structure-function relationships of immunostimulatory polysaccharides: A review. Carbohydr. Polym..

[45] Jin J.-O., Zhang W., Du J.-Y., Wong K.-W., Oda T., Yu Q. (2014). Fucoidan can function as an adjuvant in vivo to enhance dendritic cell maturation and function and promote antigen-specific T cell immune responses. PLoS ONE.

[46] Zhang W., Oda T., Yu Q., Jin J.-O. (2015). Fucoidan from Macrocystis pyrifera has powerful immune-modulatory effects compared to three other fucoidans. Mar. Drugs.

[47] Jiang Z., Okimura T., Yamaguchi K., Oda T. (2011). The potent activity of sulfated polysaccharide, ascophyllan, isolated from Ascophyllum nodosum to induce nitric oxide and cytokine production from mouse macrophage RAW264.7 cells: Comparison between ascophyllan and fucoidan. Nitric Oxide Biol. Chem..

[48] Ale M.T., Maruyama H., Tamauchi H., Mikkelsen J.D., Meyer A.S. (2011). Fucoidan from Sargassum sp. and Fucus vesiculosus reduces cell viability of lung carcinoma and melanoma cells in vitro and activates natural killer cells in mice in vivo. Int. J. Biol. Macromol..

[49] Fan Z., Wu C., Chen M., Jiang Y., Wu Y., Mao R., Fan Y. (2022). The generation of PD-L1 and PD-L2 in cancer cells: From nuclear chromatin reorganization to extracellular presentation. Acta Pharm. Sin. B.

[50] Yu X., Li W., Young K.H., Li Y. (2021). Posttranslational Modifications in PD-L1 Turnover and Function: From Cradle to Grave. Biomedicines.

[51] Li P., Bademosi A.T., Luo J., Meunier F.A. (2018). Actin Remodeling in Regulated Exocytosis: Toward a Mesoscopic View. Trends Cell Biol..

[52] Porat-Shliom N., Milberg O., Masedunskas A., Weigert R. (2013). Multiple roles for the actin cytoskeleton during regulated exocytosis. Cell. Mol. Life Sci..

[53] Aseervatham J. (2020). Cytoskeletal Remodeling in Cancer. Biology.

[54] Suresh R., Diaz R.J. (2021). The remodelling of actin composition as a hallmark of cancer. Transl. Oncol..

[55] Datta A., Deng S., Gopal V., Yap K.C.-H., Halim C.E., Lye M.L., Ong M.S., Tan T.Z., Sethi G., Hooi S.C. (2021). Cytoskeletal Dynamics in Epithelial-Mesenchymal Transition: Insights into Therapeutic Targets for Cancer Metastasis. Cancers.

[56] Ong M.S., Deng S., Halim C.E., Cai W., Tan T.Z., Huang R.Y.-J., Sethi G., Hooi S.C., Kumar A.P., Yap C.T. (2020). Cytoskeletal Proteins in Cancer and Intracellular Stress: A Therapeutic Perspective. Cancers.

[57] Kwack K.H., Ji J.Y., Park B., Heo J.S. (2022). Fucoidan (*Undaria pinnatifida*)/Polydopamine Composite-Modified Surface Promotes Osteogenic Potential of Periodontal Ligament Stem Cells. Mar. Drugs.

[58] Wu S.-Y., Chen Y.-T., Tsai G.-Y., Hsu F.-Y., Hwang P.-A. (2020). Protective Effect of Low-Molecular-Weight Fucoidan on Radiation-Induced Fibrosis through TGF-β1/Smad Pathway-Mediated Inhibition of Collagen I Accumulation. Mar. Drugs.

[59] Mustafa S., Pawar J.S., Ghosh I. (2021). Fucoidan induces ROS-dependent epigenetic modulation in cervical cancer HeLa cell. Int. J. Biol. Macromol..

[60] Wang H., Han X., Xu J. (2020). Lysosome as the Black Hole for Checkpoint Molecules. Adv. Exp. Med. Biol..

[61] Hu Y., Zhang R., Chen G. (2020). Exosome and Secretion: Action On?. Adv. Exp. Med. Biol..

[62] Xing C., Li H., Li R.-J., Yin L., Zhang H.-F., Huang Z.-N., Cheng Z., Li J., Wang Z.-H., Peng H.-L. (2021). The roles of exosomal immune checkpoint proteins in tumors. Mil. Med. Res..

[63] Oliveira R.M., Câmara R.B.G., Monte J.F.S., Viana R.L.S., Melo K.R.T., Queiroz M.F., Filgueira L.G.A., Oyama L.M., Rocha H.A.O. (2018). Commercial Fucoidans from Fucus vesiculosus Can Be Grouped into Antiadipogenic and Adipogenic Agents. Mar. Drugs.

[64] Courtois J. (2009). Oligosaccharides from land plants and algae: Production and applications in therapeutics and biotechnology. Curr. Opin. Microbiol..

[65] Gupta D., Silva M., Radziun K., Martinez D.C., Hill C.J., Marshall J., Hearnden V., Puertas-Mejia M.A., Reilly G.C. (2020). Fucoidan Inhibition of Osteosarcoma Cells is Species and Molecular Weight Dependent. Mar. Drugs.

[66] Hsu H.-Y., Hwang P.-A. (2019). Clinical applications of fucoidan in translational medicine for adjuvant cancer therapy. Clin. Transl. Med..

[67] Kwak J.-Y. (2014). Fucoidan as a marine anticancer agent in preclinical development. Mar. Drugs.

[68] Park A.Y., Nafia I., Stringer D.N., Karpiniec S.S., Fitton J.H. (2021). Fucoidan Independently Enhances Activity in Human Immune Cells and Has a Cytostatic Effect on Prostate Cancer Cells in the Presence of Nivolumab. Mar. Drugs.

[69] Zhang W., Hwang J., Yadav D., An E.-K., Kwak M., Lee P.C.-W., Jin J.-O. (2021). Enhancement of Immune Checkpoint Inhibitor-Mediated Anti-Cancer Immunity by Intranasal Treatment of Ecklonia cava Fucoidan against Metastatic Lung Cancer. Int. J. Mol. Sci..

[70] An E.-K., Hwang J., Kim S.-J., Park H.-B., Zhang W., Ryu J.-H., You S., Jin J.-O. (2022). Comparison of the immune activation capacities of fucoidan and laminarin extracted from Laminaria japonica. Int. J. Biol. Macromol..

[71] Groult H., Cousin R., Chot-Plassot C., Maura M., Bridiau N., Piot J.-M., Maugard T., Fruitier-Arnaudin I. (2019). λ-Carrageenan Oligosaccharides of Distinct Anti-Heparanase and Anticoagulant Activities Inhibit MDA-MB-231 Breast Cancer Cell Migration. Mar. Drugs.

