# Supplementary material for: Anti-Proliferative and Pro-Apoptotic vLMW Fucoidan Formulas Decrease PD-L1 Surface Expression in EBV Latency III and DLBCL Tumoral B-Cells by Decreasing Actin Network
Source: Mar Drugs. 2023 Feb 18;21(2):132. doi: 10.3390/md21020132 (PMC9963441; doi:10.3390/md21020132)
Supplement: Supplementary file 1 [file marinedrugs-21-00132-s001.zip › marinedrugs-2211828-supplementary.pdf]

Article – Supplemental data

Anti-proliferative and pro-apoptotic vLMW fucoidan formulas decrease PD-L1 surface expression in EBV latency III and DLBCL tumoral B-cells by decreasing actin network

Jennifer Saliba [1], Chanez Manseur [2], Hugo Groult [2], Hussein Akil [1], Mona Tannoury [3], Danielle Troutaud [1], Thierry Maugard [2], Jean Feuillard [1,4], Ingrid Arnaudin [2] and Chantal Jayat-Vignoles [1]

[1] UMR CNRS 7276, INSERM 1262, Laboratoire CRIBL, Faculté de Médecine, Université de Limoges, France

[2] UMR CNRS 7266, Laboratoire LIENSs, Université de La Rochelle, France;
[3] Faculté des Sciences II, Université Libanaise, Liban
[4] Laboratoire d'Hématologie, CHU Dupuytren, 2 Avenue Martin Luther King, 87042 Limoges Cedex, France

* Correspondence: chantal.vignoles@unilim.fr; Tel.: 33 (0) 5 19 56 42 21; Fax: 33 (0) 5 55 43 58 97)

Table S1. Antibodies used for flow cytometry

PE: Phycoerythrin – APC: Allophycocyanin

Specificity	Clone	Isotypic control	Clone	Fluorochrome	Supplier	Dilution
CD19	HIB19	IgG1	MOPC-21	APC	Biolegend	1/10
PD-L1 (CD274)	29E.2A3	IgG2b	MPC-11	PE	Biolegend	1/10

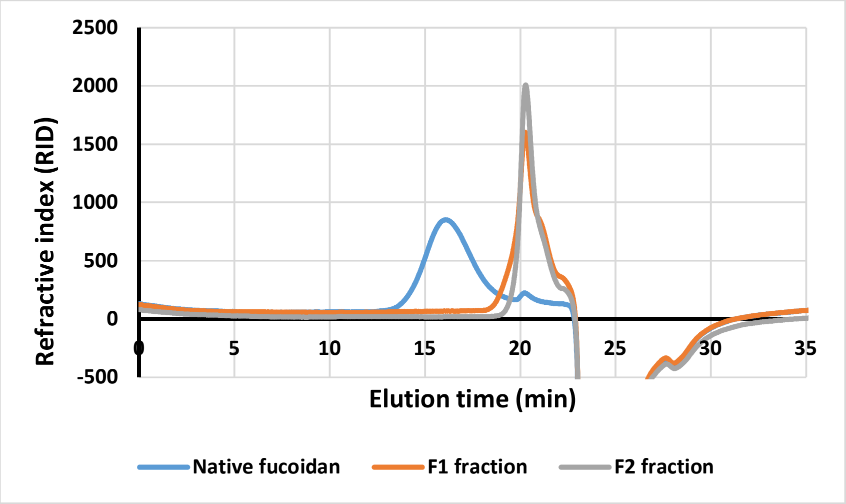

Figure S1. SEC-HPLC analysis with refractive detector of native fucoidan with F1 and F2 produced fractions

The SEC (size exclusion chromatography) separation was performed on a TSK-GEL G4000PW column in series with a TSK-GEL G3000PWXL at a flow rate of 0.8 mL/min using 0.1 M sodium nitrate ($NaNO_3$) as the eluent.

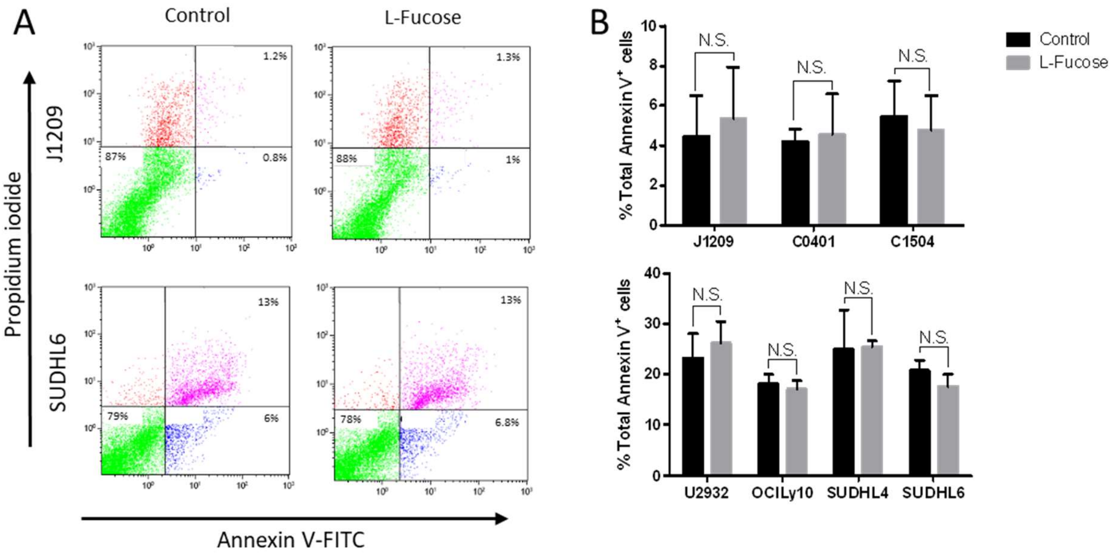

Figure S2. L-fucose does not induce apoptosis in LCLs and DLBCLs

LCLs (J1209, C0401, C1504) and DLBCLs (U2932, OCILy10, SUDHL4, SUDHL6) cells were treated or not (control) with 100µg/mL of L-fucose for 48h, followed by apoptosis analysis (Annexin V/PI staining) by flow cytometry. Results were obtained from three independent experiments.

(A) Examples of cell apoptosis for J1209 (LCL) and SUDHL6 (DLBCL) are shown (intact cells: green events – early apoptotic cells: blue events – late apoptotic cells: purple events). (B) Percentage of LCLs or DLBCLs total Annexin V^+ cells. L-fucose does not induce apoptosis in all cell lines.

NS: Not significant; * p< 0.05; **p<0.01; ***p<0.001; ****p<0.0001.

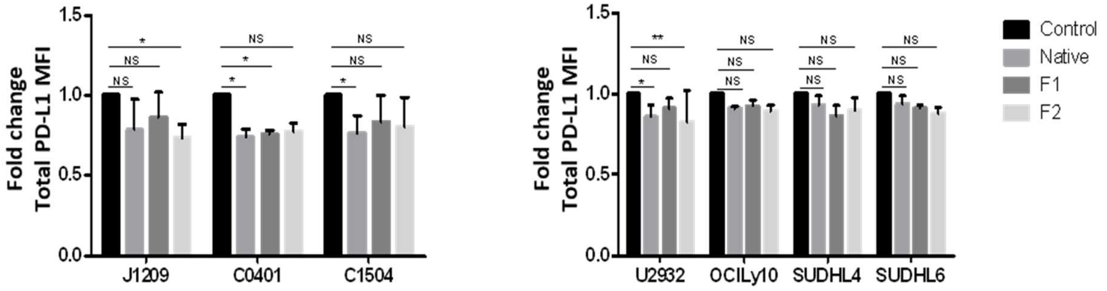

Figure S3. Native fucoidan or vLMW-F do not (or slightly) change PD-L1 total expression

LCLs (J1209, C0401, C1504) and DLBCLs (U2932, OCILy10, SUDHL4, SUDHL6) cells were treated or not (control) with 100µg/mL of native form or vLMW-F. Total (membrane and intracellular) expression of PD-L1 was assessed by flow cytometry and represented by the fold change (Ratio of MFI test/MFI control, both normalized to the isotypic control – MFI: Mean Fluorescence Intensity). No remarkable change was observed for PD-L1 total expression.

NS: Not significant; * p< 0.05; **p<0.01; ***p<0.001; ****p<0.0001.

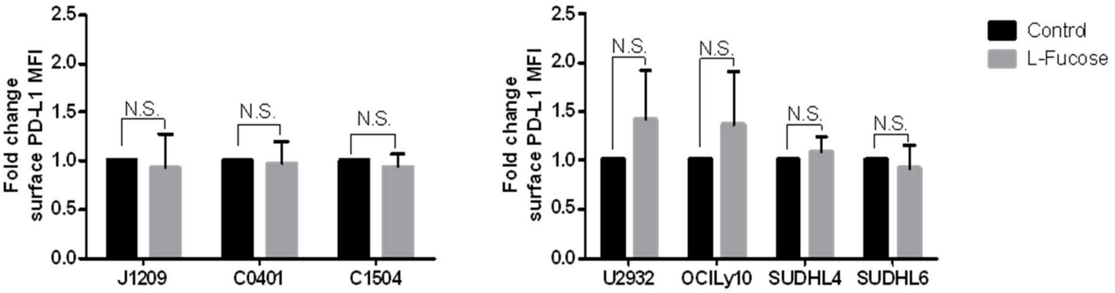

Figure S4. L-fucose does not decrease PD-L1 surface expression in LCLs and DLBCLs cells

LCLs (J1209, C0401, C1504) and DLBCLs (U2932, OCILy10, SUDHL4, SUDHL6) cells were treated with 100µg/mL of L-fucose for 48h, followed by immunofluorescent staining for PD-L1 analyzed by flow cytometry. Results were obtained from three independent experiments.

Fold change (Ratio of MFI test/MFI control, both normalized to the isotypic control – MFI: Mean Fluorescence Intensity) of PD-L1 surface expression for LCLs and DLBCLs was not decreased in the presence of L-fucose.

NS: Not significant; * $p < 0.05$; ** $p<0.01$; *** $p<0.001$; **** $p<0.0001$.
